# Improving the Efficiency of Inferences From Hybrid Samples for Effective Health Surveillance Surveys: Comprehensive Review of Quantitative Methods

**DOI:** 10.2196/48186

**Published:** 2024-03-07

**Authors:** Mansour Fahimi, Elizabeth C Hair, Elizabeth K Do, Jennifer M Kreslake, Xiaolu Yan, Elisa Chan, Frances M Barlas, Abigail Giles, Larry Osborn

**Affiliations:** 1 Marketing Systems Group Horsham, PA United States; 2 Truth Initiative Schroeder Institute Washington, DC United States; 3 Ipsos USA San Francisco, CA United States

**Keywords:** hybrid samples, composite estimation, optimal composition factor, unequal weighting effect, composite weighting, weighting, surveillance, sample survey, data collection, risk factor

## Abstract

**Background:**

Increasingly, survey researchers rely on hybrid samples to improve coverage and increase the number of respondents by combining independent samples. For instance, it is possible to combine 2 probability samples with one relying on telephone and another on mail. More commonly, however, researchers are now supplementing probability samples with those from online panels that are less costly. Setting aside ad hoc approaches that are void of rigor, traditionally, the method of composite estimation has been used to blend results from different sample surveys. This means individual point estimates from different surveys are pooled together, 1 estimate at a time. Given that for a typical study many estimates must be produced, this piecemeal approach is computationally burdensome and subject to the inferential limitations of the individual surveys that are used in this process.

**Objective:**

In this paper, we will provide a comprehensive review of the traditional method of composite estimation. Subsequently, the method of composite weighting is introduced, which is significantly more efficient, both computationally and inferentially when pooling data from multiple surveys. With the growing interest in hybrid sampling alternatives, we hope to offer an accessible methodology for improving the efficiency of inferences from such sample surveys without sacrificing rigor.

**Methods:**

Specifically, we will illustrate why the many ad hoc procedures for blending survey data from multiple surveys are void of scientific integrity and subject to misleading inferences. Moreover, we will demonstrate how the traditional approach of composite estimation fails to offer a pragmatic and scalable solution in practice. By relying on theoretical and empirical justifications, in contrast, we will show how our proposed methodology of composite weighting is both scientifically sound and inferentially and computationally superior to the old method of composite estimation.

**Results:**

Using data from 3 large surveys that have relied on hybrid samples composed of probability-based and supplemental sample components from online panels, we illustrate that our proposed method of composite weighting is superior to the traditional method of composite estimation in 2 distinct ways. Computationally, it is vastly less demanding and hence more accessible for practitioners. Inferentially, it produces more efficient estimates with higher levels of external validity when pooling data from multiple surveys.

**Conclusions:**

The new realities of the digital age have brought about a number of resilient challenges for survey researchers, which in turn have exposed some of the inefficiencies associated with the traditional methods this community has relied upon for decades. The resilience of such challenges suggests that piecemeal approaches that may have limited applicability or restricted accessibility will prove to be inadequate and transient. It is from this perspective that our proposed method of composite weighting has aimed to introduce a durable and accessible solution for hybrid sample surveys.

## Introduction

The survey sampling landscape is rapidly evolving. In an era of diminishing response rates and escalating costs, more effective survey sampling alternatives are no longer academic curiosities [1]. While the new realities suggest that departures from traditional methods are becoming inevitable, they also beckon an immediate question as survey researchers continue to experiment with hybrid sampling techniques. That is,

Are such sampling alternatives conducive to the inferential integrity of scientific surveys by reaching a representative subset of the target population in a pragmatic and cost-effective manner?

In addition to adopting multiple modes of data collection [2] it has become a customary practice to use less expensive samples selected from online panels to supplement costly alternatives from address or telephone frames [3]. The so-called opt-in panels are compiled using a potpourri of recruitment techniques, mostly relying on social media to fish for individuals willing to partake in surveys—hence the term river sampling [4]. The resulting convenience of these recruitment methods, however, is often achieved at the expense of compromising the organic representation that has been a natural byproduct of probability-based samples. This trade-off becomes of elevated concern since with samples obtained from opt-in panels, typical geodemographic weighting adjustments may no longer be adequate for ensuring their representativity [5].

It has been suggested that with such samples more granular weighting and calibration adjustments become necessary to ameliorate their compromised representations [6]. Specifically, calibration adjustments to behavioral and attitudinal benchmarks that go beyond geodemographic corrections might be needed to improve the representation of survey respondents from less representative samples [7,8]. Moreover, when mixing samples secured from different sampling frames, special procedures must be used to combine the various sample components in an optimal fashion [9]. If conducted effectively, the resulting hybrid samples may address both cost and coverage challenges of traditional single-frame sample surveys—especially when surveying rare or hard-to-get cohorts.

While the literature on how to improve the external validity of survey estimates from nonprobability samples is maturing, existing studies have focused solely on surveys of adults [10-12]. Moreover, proposed methods are often theoretical in nature or pertain to ad hoc techniques with limited scalability. Given the increasing inefficiencies of traditional survey sampling methods on the one hand, and the growing possibilities of emerging alternatives on the other, it is incumbent upon data scientists to explore innovative options that can address the evolving challenges facing the survey research community. This includes being able to produce useful inferences even when working with less-than-perfect data [13].

In this paper, we present practical weighting and calibration techniques that can be used to address the unique nuances of hybrid samples with varying representational properties, including those for the surveys of hard-to-get cohorts such as young adults. We will start with a review of the classical statistical technique known as composite estimation for combining survey estimates from 2 samples [14,15]. Next, as an extension of the methodology developed by Fahimi [16], we will introduce the method of composite weighting that is significantly more efficient, both computationally and inferentially, when pooling data from multiple surveys. For empirical illustrations, we will demonstrate results using data from 3 surveys with hybrid samples composed of probability-based components from the United States Postal Service address database and supplemental samples from online panels.

## Methods

### Ethical Considerations

All study procedures were conducted in accordance with the Declaration of Helsinki and its amendments. All study participants provided informed consent prior to being included in the study, which was approved by Advarra Institutional Review Board (IRB protocol numbers: Pro00010120 and Pro00009087).

### Mathematics of Survey Data Integration

As mentioned earlier, there is a growing interest in hybrid methodologies that combine 2 or more independent samples with varying representational properties to reduce cost. This includes combining probability samples that could be based on random digit dialing or address-based sampling, as well as the many instances where probability and nonprobability samples are combined. Integrating survey data from independent samples provides a larger analytical database with enhanced inferential possibilities.

Data pooling is also relevant to regional surveys that are conducted independently of their national counterparts, but in which both surveys collect similar data from a common cohort. In these situations, one might be interested in combining data from a regional survey with those obtained from the corresponding subset of the national survey. For example, the National Health Interview Survey [17] and the Behavioral Risk Factor Surveillance System [18] both have national as well as local components that can be combined to produce more robust estimates at overlapping domains.

While the various algebraic building blocks of composite estimation methodology have been referenced in different textbooks and papers, to the best of our knowledge, the entire inferential machinery underpinning this cumbersome process has never been furnished in full detail in 1 place. As such, in the following section, we will provide a comprehensive description of the mathematics of this classical methodology under varying scenarios.

### Composite Estimation Methodology

Traditionally, the method of composite estimation has been used to blend results from different surveys to improve the robustness of the resulting estimates [19]. That is, individual point estimates from different surveys are produced and then blended together into 1 estimate at a time. In this section, we will furnish the mathematical foundation for this arduous approach before a more efficient alternative is introduced that can produce more stable estimates while significantly reducing the computational burden.

Consider a population of *N* units from which 2 independent samples of size *n*_1_ and *n*_2_ have been selected. Under the conventional composition methodology, individual estimates from the 2 samples are produced separately and then combined to produce composite estimates that might be more robust. When the parameter of interest is, say population mean 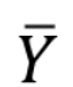, the general composite estimator will have the following form [20]:



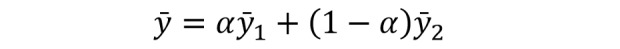

**(1)**


In the above equation, 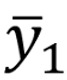 and 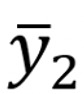 represent independent estimates of 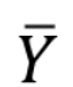 from the first and second samples, respectively. Using this decomposition, the optimal value for the blending or composition factor *α* can be obtained by minimizing the mean square error of 
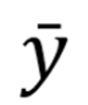
, which is a function of the variance and bias of this composite estimate [21]:



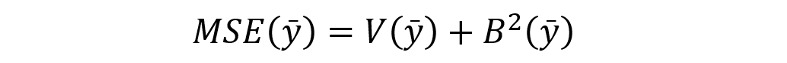

**(2)**


Under this general scenario when neither of the 2 estimates can be considered unbiased, the optimal value of *α* can be obtained by [22]:



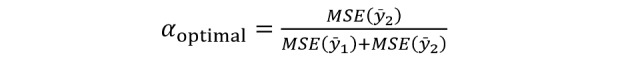

**(3)**


As mentioned earlier, a growing number of surveys complement their main probability samples with less expensive supplements secured from online panels from which the resulting estimates may not be unbiased. When probability and nonprobability samples are to be combined whereby only 1 of the 2 samples can provide unbiased estimates, say when 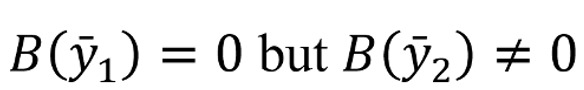 the optimal value of *α* becomes:



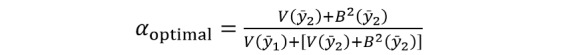

**(4)**


However, when the 2 independent estimates 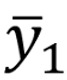 and 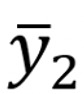 could be assumed to have a negligible bias due to the application of survey weights, then the optimal value of α can be obtained by simply minimizing the variance of 
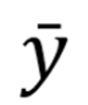
:



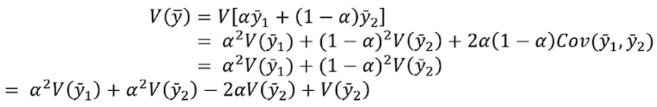

**(5)**


The minimum of the above quadratic function of *α* is that value for which its derivative is equal to 0, that is:



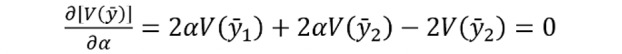

**(6)**


Consequently,



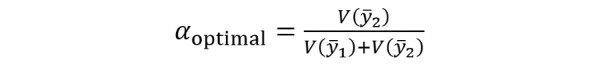

**(7)**


Furthermore, when estimates from the 2 surveys are expected to exhibit comparable variabilities as well, the above becomes a simpler function in terms of the sample sizes *n*_1_ and *n*_2_ and their associated unequal weighting effects (UWE) 
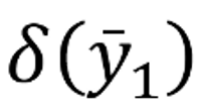
 and 
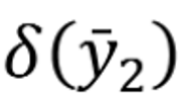
. In this case, the optimal value of the composition factor can be obtained by [16]:



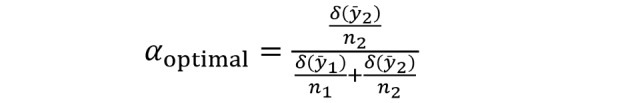

**(8)**


Finally, there are situations where it would be justifiable to assume that 
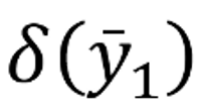
 and 
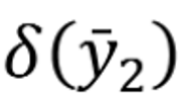
 ratio to near unity. In such instances, the optimal value of *α* reduces to a basic function of the respective sample sizes of the 2 surveys:



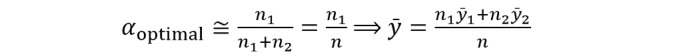

**(9)**


Whether any of the above simplifying assumptions can be justified or not, the fact remains that the composite estimation methodology entails operational complexities and inferential inefficiencies. First, this burdensome approach requires that composite estimates be produced 1 estimate at a time. Given that for a typical survey one must produce dozens of estimates for key outcome measures, this computationally intensive methodology requires serious time and resources.

Second, and more importantly, this piecemeal process produces estimates that are based on individual samples of size *n*_1_ and *n*_2_, and not the larger combined sample of size *n* = *n*_1_ + *n*_2_. This means relying on 2 estimates that could have been created using different methodologies with weighting adjustment granularities that will be coarser than what would be possible with a larger combined sample. In the next section, we will introduce an alternative methodology that can bypass these inefficiencies and complexities.

### Composite Weighting Methodology

As described above, the classical composite estimation methodology is both cumbersome and inferentially inefficient. Our proposed methodology detailed here eliminates the above inefficiencies and complexities by allowing the 2 samples to be integrated first so that a single set of composite weights could be generated for estimation purposes. Specifically, instead of producing composite estimates from the unintegrated survey data 1 at a time, under this alternative, a single set of weights will be generated so that estimates could be produced from the combined sample. This huge convenience also benefits from all the inferential dividends the larger combined sample can offer. While the following derivation is for integrating data from 2 surveys, as demonstrated later, this scalable approach can easily apply when more than 2 surveys are involved.

For ease of illustration and without the loss of generality, we can assume there is only 1 weighting cell for poststratification purposes, and let

*B*_1_*_i_*: Sampling base weights from sample 1, *i*=1,..., *n*_1_

*B*_2_*_j_*: Sampling base weights from sample 2, *j*=1,..., *n*_2_

Based on the conventional composition method, separately poststratified weights for the 2 samples will have the following form:



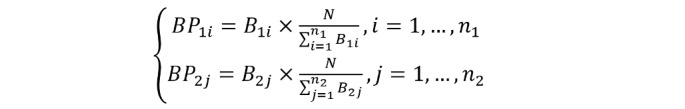

**(10)**


But rather than computing separate point estimates using the above 2 sets of poststratified weights and then compositing them 1 at a time, if the condition in equation 9 holds, one can create component weights that could be combined to aggregate to the same population total *N*. The resulting weights can then be used to produce point estimates directly from the combined data without any need for piecemeal compositions. These weights could be produced by:



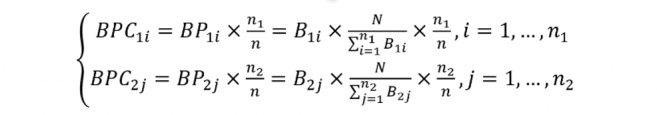

**(11)**


Given that the above approach still requires separate poststratification of individual samples, it would be desirable if the 2 sets of base weights could be combined first and then poststratified jointly. This is a vastly superior option because it can accommodate more consistent and granular weighting adjustments, courtesy of the larger combined sample. Mathematically, this can be accomplished by a simple rescaling of individual base weights first and then combining them for a join poststratification or raking by:



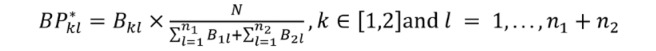

**(12)**


The above, however, magnifies the respondents’ base weights across the 2 samples with the same poststratification factor irrespective of any differential precision that would be associated with the sample that has better representation. The procedure described next introduces a simple calibration adjustment that could be used to remove this inequity prior to a joint poststratification.

### Calibration of Base Weights for Joint Poststratification

In order for the alternative weighting procedure to produce final weights that are identical to the composite weights, the following must hold:



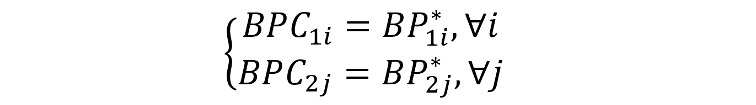

**(13)**


Specifically, the above conditions would hold if the following were satisfied:



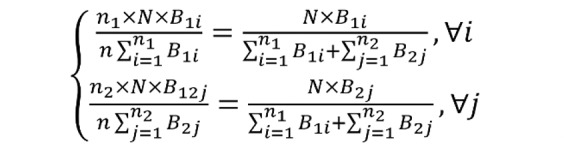

**(14)**


This means that the alternative method can produce the same composite weights, provided that base weights from the 2 samples are calibrated prior to poststratification. That is when base weights from the 2 samples are first scaled to their respective sample sizes. Having done this, instead of separately poststratifying base weights from the 2 samples and then producing composite weights, one can use the proposed calibrated base weights from the 2 samples so that the 2 can be combined and poststratified concurrently.

It should be noted that the above calibration correction easily carries over to more realistic situations with more than 1 poststrata, where the underlying assumption in equation 9 is easier to satisfy. Also, one can apply the above procedure under the less restrictive condition in equation 8 when the UWE do not ratio to unity. In this more realistic situation, the corresponding base weights must be calibrated to their respective effective sample sizes as shown:



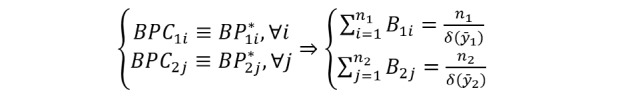

**(15)**


Estimates of survey-specific UWE are often readily available, or they can be quickly approximated as a function of poststratified (or base) weights by the following formula when finite population corrections can be ignored [23].



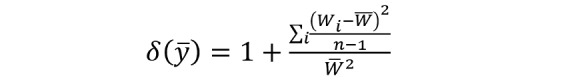

**(16)**


With the above correction applied, it would then be possible to use the resulting calibrated base weights as input for a final poststratification or raking of the combined sample using an expanded set of benchmarks. Alternatively, the same benchmarks could be used but with more granularity to improve the representation of the combined sample with respect to finer categories of the weighting variables.

Of note, the above approximation for UWE in equation 16 can also be used to assess the variability of estimates across surveys. Recognizing that variance is an estimate-specific statistic and mostly influenced by sample size and dispersion of the moments of the given estimate, UWE can be used as a relative measure of variability at the survey level as well. Typically, larger values of UWE are indicative of less representative sample surveys for which more variable weights have been needed to realign respondents vis-à-vis their population benchmarks.

### Extension and Generalization

An extension of the above applies to instances where more than 2 surveys are to be integrated. In such situations, the existing weights for each survey can be calibrated by the following optimal composition factors to produce the final blended weights for combining *S* different samples:



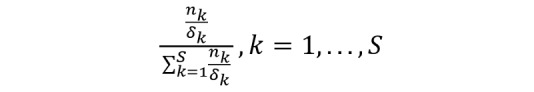

**(17)**


It is worth noting when large samples from opt-in panels are used to supplement probability samples of modest size since selection probabilities for such samples are not available, typically they are assigned a constant pseudo design weight prior to poststratification. An implication of this shortcut is that nonprobability sample components can carry artificially smaller UWE, which means such sample components will have inflated contributions when surveys are integrated. However, partitioning the total UWE for the probability sample component into that due to design weights and the residual due to poststratification can address this problem. The residual UWE for the probability sample component, which will be used for calibration correction in equation 15 will be:



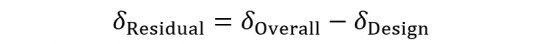

**(18)**


## Results

To illustrate the applications of our proposed methodology, data from 3 pairs of surveys were used. Each pair was comprised of data from the Truth Longitudinal Cohort (TLC) survey and the Truth’s Continuous Tracker Online (CTO) survey. The TLC is a national representative panel of youths and young adults (aged 15-24 years) recruited via address-based sampling with online data collection. The CTO is a weekly, cross-sectional survey of participants aged 15-24 years. The CTO surveys are conducted using a sample of approximately 300 respondents per week from the national Dynata online panel. Participants of the TLC and CTO are invited to complete short online surveys about attitudes, beliefs, experimentation, and frequency of use regarding tobacco and other substances.

For the first pair, the CTO component included weekly surveys from July 20, 2020, to November 17, 2020. For the second pair, these weekly samples span from July 18, 2020, to February 24, 2021. For the third pair, the CTO component was comprised of weekly samples from July 14, 2021, to September 15, 2021. For each pair, the TLC component included surveys conducted during contemporaneous time periods. Table 1 provides a summary of the 3 pairs of TLC and CTO surveys used for this research.

For this investigation, we focused on 2 key outcome parameters, prevalence of current use (eg, at least 1 day of the past 30 days) of cigarettes and e-cigarettes. Prevalence estimates were produced separately from each sample component of each survey for respondents 15 to 17, 18 to 24, and 15 to 24 years of age after each component was weighted to basic geodemographic benchmarks of the given cohort.

Table 2 provides a summary of weighted point estimates for each survey and cohort, while Table 3 shows the corresponding overall estimates for 15- to 24-year-olds. All estimates are accompanied by their associated lower confidence limit and upper confidence limit at 95% CI, for which SEs were estimated using the method of Taylor Series Linearization in SAS [24].

To compare survey estimates produced under our proposed method of composite weighting against those using composite estimation, first the above point estimates had to be combined using the latter method. That is, point estimates from individual surveys were blended one by one using the coarse weighting methodology each sample survey could tolerate. Table 4 provides a summary of the resulting composite estimates by survey pair, cohort, and outcome measure.

Subsequently, composite weights were computed for each sample pair using our proposed methodology outlined above. These weights were computed for the combined TLC and CTO samples, where more granular weighting adjustments were possible due to larger sample sizes. Moreover, additional calibration adjustments were applied to the combined sample for which the needed benchmarks were generated from the TLC sample component for each survey pair. These adjustments included corrections with respect to the following behavioral attributes, which were shown to differentiate between young adult respondents from online panels and their cohorts. (1) Length of residence: about how long have you lived at your current address? (2) Household (dwelling) type: which of the following best describes your home? (3) Financial comfort: how would you describe your family’s overall financial situation? (4) Living with parents: do you currently live in a household with at least 1 of your parents? (5) Social media influencer: do you like to be a social media influencer?

While the rationale for the above calibration adjustments for general population surveys is detailed in [6,10], for the CTO surveys further investigations were carried out to identify differentiating attributes unique to teens and young adults. It is worth noting that for instances when a probability sample component like the TLC is not available in parallel, it is possible to use government sources, such as the monthly Current Population Survey or the American Community Survey, to secure relevant benchmarks for calibration adjustments.

It is of particular importance to note that the above calibration adjustments would not have been possible under the traditional composition methodology, whereby estimates are generated from individual surveys and then combined. Table 5 provides a summary of the resulting estimates produced by integrating the TLC and CTO sample components first and then weighting them to a more granular set of benchmarks and calibration adjustments listed above.

While independent estimates of tobacco use behaviors among teens and young adults can vary greatly due to methodological differences between their corresponding surveys, the following statistics from the National Youth Tobacco Survey [25] conducted by the Centers for Disease Control and Prevention were used to assess the external validity of estimates produced using our proposed methodology. Confounding differences among surveys could be due to study design and mode of administration, as well as other differences in periodicity and questionnaire wording. Cognizant of these differences, nonetheless, our estimates using composite weighting methodology produce comparable estimates to those from the National Youth Tobacco Survey 2020, as summarized in Table 6.

**Table 1:** Sample size summary for the TLC^a^ and CTO^b^ sample components by survey pair.

Survey and cohort		Pair 1, n	Pair 2, n	Pair 3, n	Total, N
**TLC**					
	15-17	998	422	555	1975
	18-24	2363	1019	1229	4611
	15-24	3361	1441	1784	6586
**CTO**					
	15-17	1041	1112	857	3010
	18-24	2747	2876	2113	7736
	15-24	3788	3988	2970	10,746
**TLC+CTO**					
	15-17	2039	1534	1412	4985
	18-24	5110	3895	3342	12,347
	15-24	7149	5429	4754	17,332

^a^TLC: Truth Longitudinal Cohort.

^b^CTO: Continuous Tracker Online.

**Table 2:** Point estimates and confidence limits by outcome measures, survey pair, and cohort.

Survey pair and outcome				15 to 17-year-old				18 to 24-year-old		
				Sample, n		(%), estimate (95% CI)		Sample, n		(%), estimate (95% CI)
**Pair 1**										
	**TLC^a^**			998				2363		
		Cigarette			2.2 (1.3-3.1)				6.7 (5.5-7.9)	
		e-Cigarette			8.2 (6.2-10.2)				15.0 (13.2-16.7)	
	**CTO^b^**			1041				2747		
		Cigarette			5.5 (3.7-7.4)				22.1 (19.9-24.2)	
		e-Cigarette			10.4 (7.9-12.9)				25.5 (23.1-27.9)	
**Pair 2**										
	**TLC**			422				1019		
		Cigarette			1.2 (0.1-2.2)				6.7 (4.8-8.6)	
		e-Cigarette			7.0 (4.5-9.5)				17.4 (14.6-20.3)	
	**CTO**			1112				2876		
		Cigarette			6.1 (4.4-7.9)				22.3 (20.2-24.5)	
		e-Cigarette			14.0 (11.4-16.6)				27.5 (25.1-29.9)	
**Pair 3**										
	**TLC**			555				1229		
		Cigarette			3.0 (1.4-4.7)				6.9 (5.2-8.7)	
		e-Cigarette			5.7 (3.6-7.8)				18.1 (15.5-20.7)	
	**CTO**			857				2113		
		Cigarette			5.7 (3.9-7.6)				25.0 (22.3-27.7)	
		e-Cigarette			12.4 (9.5-15.4)				28.8 (25.9-31.7)	

^a^TLC: Truth Longitudinal Cohort.

^b^CTO: Continuous Tracker Online.

**Table 3:** Point estimates and confidence limits by outcome measures and survey pair (15 to 24 years of age).

Survey pair and outcome				Sample size, n	(%), estimate (95% CI)
**Pair 1**					
	**TLC^a^**		3361		
		Cigarette			5.3 (4.4-6.2)
		e-Cigarette			12.9 (11.5-14.2)
	**CTO^b^**		3788		
		Cigarette			16.9 (15.3-18.5)
		e-Cigarette			20.8 (18.9-22.6)
**Pair 2**					
	**TLC**		1441		
		Cigarette			5.0 (3.6-6.3)
		e-Cigarette			14.2 (12.0-16.3)
	**CTO**		3988		
		Cigarette			17.2 (15.6-18.8)
		e-Cigarette			23.2 (21.4-25.1)
**Pair 3**					
	**TLC**		1784		
		Cigarette			5.7 (4.4-7.0)
		e-Cigarette			14.2 (12.3-16.2)
	**CTO**		2970		
		Cigarette			18.9 (16.9-20.9)
		e-Cigarette			23.6 (21.4-25.8)

^a^TLC: Truth Longitudinal Cohort.

^b^CTO: Continuous Tracker Online.

**Table 4:** Composite estimates by survey pair, cohort, and outcome measures.

Survey pair, cohort, and outcome				Composite		
				Factor (α)		Estimate, %
**Pair 1**						
	**15-17 (n=2039)**					
		Cigarette	.80		2.9	
		e-Cigarette	.61		9.1	
	**18-24 (n=5110)**					
		Cigarette	.75		10.5	
		e-Cigarette	.64		18.7	
	**15-24 (n=7149)**					
		Cigarette	.76		8.1	
		e-Cigarette	.64		15.7	
**Pair 2**						
	**15-17 (n=1534)**					
		Cigarette	.74		2.5	
		e-Cigarette	.52		10.4	
	**18-24 (n=3895)**					
		Cigarette	.56		13.6	
		e-Cigarette	.41		23.3	
	**15-24 (n=5429)**					
		Cigarette	.58		10.1	
		e-Cigarette	.43		19.4	
**Pair 3**						
	**15-17 (1412)**					
		Cigarette	.56		4.2	
		e-Cigarette	.66		8.0	
	**18-24 (3342)**					
		Cigarette	.55		22.9	
		e-Cigarette	.00		0.0	
	**15-24 (4754)**					
		Cigarette	.70		9.7	
		e-Cigarette	.57		18.3	

**Table 5:** Survey estimates using composite weights by survey pair, cohort, and outcome measure.

Survey pair, cohort, and smoker			(%), estimate (95% CI)
**Pair 1**			
	**15-17 (n=2039)**		
		Cigarette	3.6 (2.7-4.6)
		e-Cigarette	9.1 (7.6-10.6)
	**18-24 (n=5110)**		
		Cigarette	13.6 (12.5-14.7)
		e-Cigarette	19.5 (18.1-20.9)
	**15-24 (n=7149)**		
		Cigarette	10.5 (9.6-11.3)
		e-Cigarette	16.3 (15.2-17.3)
**Pair 2**			
	**15-17 (n=1534)**		
		Cigarette	4.4 (3.2-5.6)
		e-Cigarette	11.5 (9.6-13.4)
	**18-24 (n=3895)**		
		Cigarette	17.0 (15.5-18.5)
		e-Cigarette	23.9 (22.1-25.7)
	**15-24 (n=5429)**		
		Cigarette	13.0 (11.9-14.1)
		e-Cigarette	20.0 (18.6-21.4)
**Pair 3**			
	**15-17 (n=1412)**		
		Cigarette	4.4 (3.2-5.6)
		e-Cigarette	9.2 (7.4-10.9)
	**18-24 (n=3342)**		
		Cigarette	15.9 (14.4-17.5)
		e-Cigarette	23.4 (21.5-25.2)
	**15-24 (n=4754)**		
		Cigarette	12.3 (11.2-13.5)
		e-Cigarette	18.9 (17.5-20.3)

**Table 6:** Prevalence of cigarette and e-cigarette use among middle and high school students.

School cohort	Past 30-day use	
	Cigarette, %	e-Cigarette, %
Middle school (n=7042)	1.2-2.2	3.6-6.0
High school (n=7453)	3.6-6.0	17.2-22.2
Middle and high school (n=14,531)	2.6-4.2	11.3-15.0

## Discussion

### Principal Findings

Conducting credible survey research in the 21st century is an endeavor subject to evolving challenges that require thinking outside of the traditional survey sampling toolbox. The proliferation of such challenges has had 2 distinct impacts on survey sampling. On the one hand, there are emerging improvisational methods of sampling and weighting that, while expedient, are void of scientific underpinnings. On the other, there is a growing disenchantment with traditional methods that despite their complex and computationally intensive nature, struggle with the new realities of the digital age. Our proposed method of composite weighting addresses both deficiencies, by offering an accessible approach that is soundly grounded in inferential sciences.

The statistical machinery, survey researchers have relied upon for decades, made it possible to make measurable inferences about target populations when samples of modest size are selected from complete sampling frames; all sampling units carry known and nonzero selection probabilities; and surveys achieve near-perfect rates of response [26]. For various reasons, but most notably the growing rates of nonresponse and survey costs, many of the surveys conducted these days struggle to fulfill the fundamental tenets of this sampling paradigm [27-29]. While such violations have been commonplace and routinely discounted by market researchers for whom theoretical considerations are often trumped by cost and time constraints, arguably, even large-scale government surveys are no longer exempt from such challenges [30].

A strategy that is gaining popularity for dealing with the rising costs and coverage challenges of surveys is to combine 2 or more independent samples selected from separate sampling frames with varying representations of the target population. In particular, such hybrid alternatives can pay considerable dividends when survey data secured from certain sample components are significantly less costly. There are also instances when multiple samples are required for the design of a study, while in other situations existing data from different surveys are pooled to address the size and analytical needs of a given research.

In comparison to the traditional method of composite estimation whereby separate estimates are combined from different surveys 1 at a time, our proposed composite weighting methodology for integrating survey data offers at least 5 distinct advantages.

First, the method of composite weighting is less cumbersome than that of composite estimation because it enables researchers to work with a single data file and not multiple sets of data and weights from unintegrated surveys.

Second, an integrated database that is larger than any of the individual sample components accommodates more nuanced weighting adjustments than what might be possible with individual surveys. This becomes especially appealing when one of the surveys is based on a small sample size, whereby coarse weighting can fail to improve the representation of its respondents.

Third, integrated survey data allows more in-depth analyses, particularly when comparisons of smaller analytical subgroups are of interest. Such deep-dive multivariate analyses are not feasible when producing separate estimates from individual surveys, some of which could be of modest size.

Fourth, related to the above, survey estimates from the resulting integrated data will be subject to smaller and consistently calculated SEs courtesy of the larger sample size and a single data set to make inferences from. Composite weighting eliminates extraneous variabilities that are inevitable under composite estimation due to the application of inconsistent weighting procedures for individual surveys, such as the use of different benchmarks, raking algorithms, and weight-trimming rules.

Finally, and perhaps most importantly, composite weighting offers a unique advantage that is of particular importance as interest in combining data from probability and nonprobability samples continues to grow. Specifically, this methodology offers the possibility of additional calibration adjustments of respondents from nonprobability samples to benchmarks that are not externally available but can be generated from the “more representative” subset of respondents.

The above advantages are particularly relevant to public health research initiatives, as they often require faster data collection turnarounds from large pools of respondents. Unlike opinion polling and commercial survey applications that typically aim for “good-enough” estimates of trends, health studies often require reliable assessments that can guide public policies. It is in this context that unreliable inferences, or those that are reliable but slow to produce, can have dire consequences or be obsolete.

Yet just like any new methodology, what we have proposed is not a panacea or free from limitations. For instance, neither this approach nor any other can produce measurable inferences from data secured from sample surveys that fail to represent their target cohort due to systematic exclusions. Another potential limitation worth mentioning is the technical sophistication the application of this methodology may require. While substantially less complicated and resource-intensive as compared to the method of composite estimation, our proposed method still requires some level of inferential acumen and know-how. Then again, working with complex surveys that entail mixing data from multiple sources is an undertaking that requires a decent level of survey sampling and inferential familiarity to begin with. If tasked with the challenge of mixing data from multiple surveys, our hope is that researchers will find the option of composite weighting more accessible and efficient.

### Concluding Remarks

Despite the growing challenges facing the survey research community, practitioners should not succumb to suboptimal practices for cost-saving purposes alone. Such unilateral guidelines have contributed to the stigma that commercial surveys have inadequate concern for rigor. On the other hand, undue allegiance to traditional methods of survey sampling can also confine researchers to inefficient practices that are losing their pragmatism. This position becomes particularly untenable when such adherences are simply for the sake of preserving the optics.

Traditional methods of survey research are becoming inefficient, both with respect to data quality and cost, begging for novel and pragmatic alternatives that do not forego rigor. It is from this perspective that we hope the methodology we have furnished in this paper could address some of such inefficiencies, enabling survey researchers to take fuller advantage of the data resources they have at their disposal. Results from the detailed comparisons we have exhibited in this paper show that composite weighting methodology is vastly less cumbersome and produces estimates that are at least as reliable as what can be produced using composite estimation.

As a final parting note we would like to reiterate that with declining response rates, surveys require progressively more comprehensive weighting adjustments to restore the representation of their respondents. As such, it is not advisable to shy away from more aggressive weighting and calibration adjustments only to keep the resulting UWE at bay [31]. Of course, this is fully cognizant of the proverbial seesaw occupied on one side by bias and variance on the other. Above all, it is imperative to retain full transparency about adopted methodologies and their potential shortfalls as we explore new possibilities for survey sampling in the digital age.

## Abbreviations

CTO: Continuous Tracker Online
TLC: Truth Longitudinal Cohort
UWE: unequal weighting effect

## Data Availability

The data sets generated and analyzed during this study are not publicly available due to their proprietary nature and privacy concerns; however, they can be made available from the corresponding author on reasonable request.
